# Biomarkers as Prognostic Predictors and Therapeutic Guide in Critically Ill Patients: Clinical Evidence

**DOI:** 10.3390/jpm13020333

**Published:** 2023-02-15

**Authors:** Rosa Méndez Hernández, Fernando Ramasco Rueda

**Affiliations:** Department of Anesthesiology and Surgical Intensive Care, University Hospital of La Princesa, 28006 Madrid, Spain

**Keywords:** biomarkers, precision medicine, sepsis, perioperative risk assessment, critical care, infection, organ failure, pulmonary congestion, systemic congestion, point of care

## Abstract

A biomarker is a molecule that can be measured in a biological sample in an objective, systematic, and precise way, whose levels indicate whether a process is normal or pathological. Knowing the most important biomarkers and their characteristics is the key to precision medicine in intensive and perioperative care. Biomarkers can be used to diagnose, in assessment of disease severity, to stratify risk, to predict and guide clinical decisions, and to guide treatments and response to them. In this review, we will analyze what characteristics a biomarker should have and how to ensure its usefulness, and we will review the biomarkers that in our opinion can make their knowledge more useful to the reader in their clinical practice, with a future perspective. These biomarkers, in our opinion, are lactate, C-Reactive Protein, Troponins T and I, Brain Natriuretic Peptides, Procalcitonin, MR-ProAdrenomedullin and BioAdrenomedullin, Neutrophil/lymphocyte ratio and lymphopenia, Proenkephalin, NefroCheck, Neutrophil gelatinase-associated lipocalin (NGAL), Interleukin 6, Urokinase-type soluble plasminogen activator receptor (suPAR), Presepsin, Pancreatic Stone Protein (PSP), and Dipeptidyl peptidase 3 (DPP3). Finally, we propose an approach to the perioperative evaluation of high-risk patients and critically ill patients in the Intensive Care Unit (ICU) based on biomarkers.

## 1. Precision Medicine and Biomarkers

Medicine is directed towards personalizing treatment based on the characteristics of patients suffering from the same disease and their different reactions to the treatments they receive. This is known as precision medicine [1], because it is understood that all treatments are personalized and that what we seek with new clinical and laboratory tools is precision in diagnosis and treatment [2].

Biomarkers are the key to precision medicine because, within what is known as the omics sciences, they are the molecules currently available [3].

Although many of them are familiar in our daily lives, such as creatinine, which plays a role in renal failure, many biomarkers are relatively new and in question, in many cases due to an erroneous approach to their positioning, in others due to ignorance, and in some due to legitimate concerns about the costs versus benefits [4].

This is why we propose the following:

- First, we outline the characteristics a biomarker should have in the context of the perioperative high-risk patient and the critical patient, and how to ensure its usefulness.
- Second, we will review the biomarkers that in our opinion can be most useful in clinical practice in the future.
- Finally, we propose an approach to the perioperative evaluation of high-risk patients and critical patients based on biomarkers.

A biomarker is a molecule that can be measured in a biological sample objectively, systematically, and accurately, whose levels indicate whether a process is normal or pathological. The ideal biomarker should, in general, be easy to measure, low in cost, and high in sensitivity and specificity, and should provide additional information for the clinical assessment [5]. To ensure its usefulness before integration into clinical practice, the progressive evaluation of each new biomarker has been proposed in six steps [6]:

(1) Proof of concept: Do levels of this new marker differ between subjects with different perioperative outcomes?
(2) Prospective validation: Does the new marker predict the likelihood of certain outcomes in prospective studies?
(3) Incremental value demonstration: Does the new marker add predictive information to standard risk markers?
(4) Clinical utility: Does modifying the values of the new marker predict a risk when changing the recommended therapy?
(5) Improved clinical outcome: Does the new risk marker improve clinical outcomes?
(6) Cost-effectiveness: Does the use of the marker improve clinical outcomes to justify the additional costs associated with its use?

All of these questions are appropriate to ask when analyzing a new biomarker to integrate into clinical practice. In fact, it is very difficult for any biomarker to fully meet all these criteria, among other reasons, because of the way in which their effects are analyzed and research is proposed [7]. Biomarkers can be used for diagnosis, assessing disease severity to stratify risk, predicting and guiding clinical decisions, and guiding treatment and the response to them. The discriminative capacity of a diagnostic test refers to its usefulness in distinguishing healthy from sick individuals. To measure this capacity, the parameter to be estimated is the AUC-ROC (area under the curve ROC), a unique value independent of the prevalence of the disease. The AUC-ROC reflects the test’s ability to discriminate individuals with and without disease across the entire range of possible cutoff points. A ROC curve plot illustrates the ratio of true positives (*y*-axis) to false positives (*x*-axis) for each cutoff point of a diagnostic test whose measurement scale is continuous [8]. Therefore, biomarkers should be analyzed using the ROC curves and looking for the Youden index, which is the point of the curve that represents the best compromise between sensitivity and specificity.

There is no particular AUC-ROC value that is considered a threshold for determining whether or not a marker is useful as a predictor. A diagnostic test is considered nondiscriminatory if its ROC curve coincides with the nondiscrimination line, which has AUC = 0.50. As the AUC of a diagnostic test approaches 1.00 (a perfect diagnostic test), the greater its discriminative capacity becomes. However, if we consider that AUC = 0.75 is halfway between non-discrimination (AUC = 0.50) and perfect discrimination (AUC = 1.00), AUC is closer to perfection than to nondiscrimination. The AUC-ROC is a sample estimator of a population parameter, so its 95% confidence interval (CI) is reflected.

The approach based on using the Youden index value as a dichotomous biomarker value in decision making is problematic if the biomarker is used to rule out in the case of a negative test (high sensitivity) or to confirm a diagnosis in the case of test positivity (high specificity). In many contexts, negative and positive probability ratios can be used to select thresholds. The 95% CI can report optimal cutoff points [9]. However, a 95% CI crossing below the 0.5 probability line may indicate a low robustness of a marker. The probability ratio is the ratio between the probability of obtaining a certain outcome in sick individuals and the probability of that same outcome in well individuals. It is therefore presented as a measure that reflects the usefulness of a diagnostic test. In addition, its value is not influenced by the prevalence of the disease, and that allows comparisons between different diagnostic tests. The results of a probability ratio for a negative test, which consists of the probability that a patient with a normal test does not really have the disease, with values between 1 and 0.6, are not considered adequate to exclude the diagnostic test, while between 0.5 and 0.1 is considered of moderate value, and a good diagnostic test [10].

There are also values derived from the AUC-ROC curve that define diagnostic characteristics that may be useful. This is the case of the positive (PPV) and negative (NPV) predictive values, which depend on the prevalence, and therefore can be applied in populations with the same prevalence. A high PPV would be adequate for a biomarker from which a specific associated treatment is derived. As we will discuss in the text, there are new biomarkers for whose deficiency there is a drug as a treatment. Having a high PPV in this context is helpful. In other scenarios, a biomarker with a high NPV is useful, since it rules out the presence of disease and therefore the need to use resources or treatments that would imply increasing the expense or the possibility of iatrogenicity. The most common example is the use of D-dimer to rule out pulmonary thromboembolism, but also biomarkers such as Pro BNP, MR-ProADM, or BIoADM, among others, with high NPV able to reasonably rule out high-risk scenarios and avoid overtreatment.

The risk of a patient does not change abruptly when moving from one figure to another a little higher in a biomarker, nor are there thresholds that cause a sudden change in risk, so dichotomization of a biomarker is biologically implausible [11]. Clinical prediction models using biomarkers are typically developed using either logistic regression or Cox regression models. The choice of variables to include in a regression model needs to take into consideration which variables may have clinical relevance and be readily available. This type of model requires rigor and validation [7].

In intensive care and in the high-risk surgical patient, interest in biomarkers increases exponentially, due to the precision medicine approach [12].

The variability of the evolution of the disease in each patient leads to models that attempt to describe phenotypes that predict the evolution—if necessary, monitoring resources or admission in the intensive care unit (ICU), response to treatment, and, more recently, the association of a biomarker with the use of a specific treatment [13].

In the context of surgery, mortality is very high, close to 4%, and in critical medicine mortality due to events such as sepsis (an example of a prevalent pathology in the ICU), is 10%, rising above 40% in septic shock [14].

All this means that phenotyping, mediated by biomarkers themselves or complementing clinical or other variables (such as hemodynamics), can help improve results [15].

In a recent review, new biomarkers are analyzed, finding thousands of references and hundreds of biomarkers, including dozens of new ones. It uses an academic, discriminative approach, based on studies with more than 300 patients [16]. In this review, we will briefly comment on those that we consider most practical from the point of view of understanding the place of the biomarker in both the current clinic and future studies.

## 2. The Best Biomarkers in Intensive and Perioperative Care

We will review a list of biomarkers that, in our opinion, are relevant and the knowledge of which we consider necessary and useful for present and future clinical practice. Dichotomous cutoff points are as already noted in the introduction, although, if necessary, these should always be put into perspective and understood within each particular clinical context.

We have summarized the main characteristics and cutoff points in Table 1.

### 2.1. Lactate: A Perfusion Biomarker

Lactate is a marker of perfusion, so it has been included in the new (2016) definition of septic shock. Having a lactate value more than 2 mmol/L is a *sine qua non* for diagnosis [17]. Lactate is a product of anaerobic metabolism. In situations of low flow or tissue hypoxia, the pyruvate used to generate energy cannot enter the Krebs cycle and is reduced to lactate. Therefore, in situations of hypoperfusion, lactate increases both by an increase in its production and by not being clarified. Lactate levels are used for the diagnosis of hypoperfusion situations and to assess the effectiveness of the treatment administered, as their levels decrease over time, which is known as “clearance” [7]. In critical illness, decreases in lactate levels after initiation of treatment are associated with better outcome. 

When there is an elevation of lactic acid, we must put it in context and first rule out obvious causes of increase, such as liver failure, seizures, brain tumor pathology, carbon monoxide poisoning, or thiamine deficiency, among others, and contextualize it with other signs of hypoperfusion such as hypotension or tachycardia. After the publication of the ANDROMEDA study [18], the prolongation of the capillary filling time has been shown to work in the context of hemodynamic resuscitation and can be an alternative or complement to lactate.

However, even recognizing that the elevation of lactate in the majority of scenarios implies a worse prognosis and a red flag, the therapeutic objective of its clearance is in question since this concept may be more complex and not always effective or plausible in the biological context of the disease, so the recommendation is to use it with caution as a goal of hemodynamic resuscitation in the short term [19].

### 2.2. C-Reactive Protein: A Classic Biomarker of Inflammation 

Inflammation is a complex and nonspecific process that involves numerous defense systems of the body. The greatest virtue of C-Reactive Protein (CRP) is also its weakness, as is its sensitivity to detecting inflammation and low specificity in numerous related conditions. However, contextualized, it is very useful in intensive and perioperative care [20]. CRP is part of the short pentraxin subfamily and was identified more than 70 years ago. It is a characteristic component of “acute phase” proteins, the synthesis of which increases dramatically in inflammatory processes. It is released by the liver in response to inflammation or tissue damage. In infectious processes, CRP has both pro-inflammatory and anti-inflammatory effects [21]. CRP can recognize and bind to pathogens and damaged cells and influence their elimination through interactions with inflammatory cells and mediating molecules.

CRP is a clinical marker frequently used to evaluate the presence of infection and sepsis and is frequently used in the diagnosis of intra-abdominal infections [22], as a marker of discrimination of patients with pneumonia and those with tracheal infections [23], as an aid to differentiate bacterial infection from viral and, in critical patients, elevated CRP values have been associated with increased risk of organ failure and mortality [24]. Elevated CRP concentrations have been successfully used as a biomarker of infection in septic patients with community-acquired pneumonia (CAP) or ventilator-associated pneumonia (VAP) and as a marker of bacterial load and appropriate antibiotic therapy.

However, when compared with other biomarkers, it is observed that it rises late, takes time to recover normal values, and rises in noninfectious processes such as autoimmune or rheumatic diseases, among others.

Normal CRP values are less than 5 mg/dL, and 10 mg/L values are considered to indicate high risk of infection or severe inflammation. One of the important values of CRP is its fast and economical measurement.

### 2.3. Troponin: The Biomarker of Myocardial Damage

Cardiac troponins I (cTnI) and T (cTnT) are components of the myocyte contractile apparatus and are expressed almost exclusively in the heart. Elevated troponin values reflect myocardial damage but do not indicate the pathophysiological mechanisms involved [25]. Elevated troponin is part of the definition of myocardial injury secondary to (1) atherosclerosis of the coronary artery and its complications leading to myocardial infarction type 1; (2) an imbalance between the supply and demand of oxygen to the myocardium, which produces type 2 infarction; and (3) a myocardial injury in the clinical context of heart and noncardiac diseases [26]. There is a new entity in which there is elevation of troponins without coronary injury, called myocardial infarction with nonobstructive coronary artery disease (MINOCA) [27].

Chronic renal failure, brain lesions, and sepsis, among others, are some noncardiac entities that occur with myocardial injury and therefore with the consequent increase in troponins. Myocardial dysfunction is a frequent complication in patients with sepsis [28] and even more when septic shock develops. It is often reversible and directly related to gravity. It can cause systolic or diastolic dysfunction of the left and right heart. Numerous mechanisms have been proposed for the elevation of troponins in sepsis, such as the imbalance of supply and demand during shock with consequent ischemia and also the harmful effect of endotoxins and cytokines on the myocyte. Normal values are less than 40 ng/L cTnT, less than 34 ng/L cTnI, and less than 14ng/L for high-sensitivity cardiac troponin T (hsTnT). Most cardiovascular complications after noncardiac surgery (NCS) occur in the first 48–72 postoperative hours; 65% of patients who suffer a perioperative myocardial infarction do not experience symptoms, in part because they are probably receiving analgesic medications that mask ischemic symptoms. However, asymptomatic myocardial infarction is associated with an increased risk of mortality at 30 days. Myocardial injury after noncardiac surgery, known by the acronym MINS [29], is characterized by elevated troponins without associated infarction clinic.

These levels for the diagnosis of MINS after NCS are a hsTnT of 20 to 65 ng/L with an absolute change of at least 5 ng/L from baseline or an hsTnT level > 65 ng/L. Among patients with abnormal baseline troponin values, MINS is considered to have occurred if there is a ≥ 20% increase in cTnI or cTnT after NCS. The VISION study by Devereaux’s research group demonstrated that the detection of an elevated level of cTnT in the postoperative period was a strong predictor of mortality after 30 days in surgical patients [30]. Although no study has established optimal cTnI thresholds for the diagnosis of MINS, there is no preference for cTnT over cTnI. Until research establishes cTnI MINS thresholds, clinicians should define elevation as any value above their laboratory’s upper 99th percentile reference limit. The management of patients with perioperative troponin elevation is unclear, but it is evident that they have an increased risk of myocardial ischemia, as a spectrum from MINOCA to MINS, or as a sign of associated cardiovascular disease or sepsis. These patients could benefit from the preoperative intensification of cardiological medication, its special surveillance in the perioperative period, or the administration of antiplatelet agents.

Recently, Devereaux’s group also reported that hsTnI levels after cardiac surgery that were associated with an increased risk of death within 30 postoperative days were substantially higher than the levels currently recommended for defining a clinically important myocardial injury or for the detection of perioperative myocardial infarction [30].

### 2.4. NT-ProBNP/BNP: Biomarkers of Heart Failure and Pulmonary Congestion

The biomarkers Brain Natriuretic Peptide (BNP) and N-terminal pro-Brain Natriuretic Peptide (NT-proBNP) are synthesized in cardiac myocytes in response to increased myocardial wall stress. BNP and NT-proBNP have been positioned with evidence and in clinical practice as cardiological biomarkers for diagnosis and prognosis in patients with heart failure and other cardiac pathologies in the nonsurgical setting [31]. Current evidence suggests that pre- and postoperative monitoring of brain natriuretic peptides may substantially improve surgical risk prediction. Most guidelines recommend its use in the stratification of high-risk patients. The Canadian Cardiovascular Society Guidelines recommend (strong recommendation, moderate evidence) the determination of NT-proBNP or BNP for the estimation of perioperative risk in NCS in all patients older than 65 and in those aged 45 to 64 when they have significant heart disease or a revised cardiac risk index (RCRI) > 1 [32]. Several prospective observational studies have evaluated with favorable results the prognostic ability of BNP and NT-proBNP to predict major cardiovascular events in NCS. A meta-analysis involving 2179 patients demonstrated that preoperative determination of NT-proBNP and/or BNP was independently associated with death or nonfatal myocardial infarction within 30 days following NCS [33]. Plasma values greater than 300 pg/mL NT-proBNP and 92 pg/mL BNP were identified as thresholds associated with increased risk. Previously, another meta-analysis had shown that elevation of natriuretic peptides increased the risk of postoperative cardiac complications (OR 19.3; CI 95% 8.5–43.7) [34]. Different surgical series in general, thoracic, and orthopedic surgery have correlated an increase in preoperative levels of brain natriuretic peptides with adverse postoperative outcomes. In our setting, we have studied NT-proBNP for perioperative risk prediction [35]. The objective was to determine the incidence of elevated serum levels of NT-ProBNP before and after major elective NCS and to evaluate its relationship with the incidence of cardiovascular complications and mortality in the first 30 postoperative days. A total of 304 adult patients with cardiovascular risk factors were recruited. The overall incidence of cardiovascular complications was 7.8% and the mortality rate was 4.3%. The presence of elevated preoperative NT-proBNP levels was an independent predictor of cardiovascular complications and mortality. Thus, for a preoperative value of >1000 pg/mL, the incidence of cardiological complications was 22.4% and mortality was 13.2%. The results highlight the high negative predictive value of NT-proBNP, with a strong association between normal preoperative values of the biomarker and a favorable outcome after NCS.

In the context of intensive care, BNP and NT-proBNP have been observed to be associated with myocardial dysfunction and right ventricular dysfunction in patients with sepsis-associated cardiomyopathy [36]. The release of BNP and NT-proBNP in these patients is stimulated by the stretching of myocytes when ventricular dysfunction is present and by proinflammatory molecules. In addition, natriuretic peptides can detect pulmonary congestion and have been used to guide treatment, considering a 30% decrease from their initial value as a therapeutic target of decongestion [37]. Proper resuscitation with fluids in the early stages of sepsis leads to better results; this often requires a delicate balance between infraresuscitation and volume overload [38]. Identifying patients sensitive to volume overload remains a major challenge. There are simple dynamic measures to identify patients who will respond by improving their cardiac output if they receive volume, but there are no simple measures to identify congestion or the possibility of diastolic dysfunction. In patients with heart failure, natriuretic peptides have been shown to be useful markers of volume status, preload, and end-diastolic volume. NT-ProBNP is considered one of the biomarkers of congestion (together with bioadrenomedullin -bioADM) in the context of acute heart failure.

The disadvantages of the use of natriuretic peptides are the difficulty of detecting systemic congestion due to right failure (peptides are produced mainly on the left side) and producing its elevation in other noncongestive circumstances such as concomitant renal failure or processes related to care such as catecholamine infusion and volume resuscitation [39].

The optimal limit of BNP and NT-proBNP for predicting mortality in sepsis remains uncertain and ranges from 32 to 681 pg/mL for BNP to 400–13,600 pg/mL for NT-ProBNP [40]. A recent meta-analysis was unable to determine optimal cutoff points for mortality and prognosis outcomes in patients with sepsis [41]. In the evaluation of patients with dyspnea, a BNP level of <100 pg/mL has been used as a sensitive and specific value to rule out heart failure.

For NT-proBNP, a cutoff point of 300 pg/mL is used to rule out heart failure [42]. The cutoff points changing with age are 450 pg/mL for <50 years, 900 pg/mL for 50–75 years, and 1800 pg/mL for >75 years old [43].

A systematic review and meta-analysis of 36 studies and 3508 patients found that BNP and NT-proBNP are frequently elevated in patients with sepsis, are prognostic in this population, and that the optimal cutoff points for BNP and NT-proBNP were calculated at 622 pg/mL and 4000 pg/mL for short-term prediction of mortality in patients with sepsis and septic shock [41]. Most studies measured biomarkers at admission or within the first 24 h.

### 2.5. Procalcitonin: The Biomarker of Infection

Procalcitonin (PCT) is a common marker in clinical practice. Its use as a potential marker of infection was first described by Spanish researchers in 1975. PCT, the calcitonin prohormone, is produced in response to the release of endotoxin or mediators released in response to bacterial infections and has a strong correlation with the severity and extent of infection [44]. Normal values are considered 0.05–0.09 ng/mL. PCT has a higher kinetic profile than CRP. It begins to rise in the first 4 to 12 h after stimulation, and its circulating levels are reduced daily by half once the infection is controlled by the host’s immune system and by antibiotic therapy.

Compared to the other available biomarkers, it is able to relatively easily discriminate infection from systemic inflammation of another origin. PCT levels also correlate with severity of infection and bacterial load. In patients with CAP or urinary tract infection (UTI), values below 0.1 ng/mL have a high sensitivity for the exclusion of bacteremia [45]. Likewise, it has the potential to differentiate the viral or bacterial origin of the infection [46], as well as to indicate the presence of bacterial superinfection in patients with viral diseases. It is interesting to note that PCT does not seem to be attenuated by the use of corticosteroids and that its production does not depend on leukocytes. Table 2 shows the most accepted reference values for the diagnosis of sepsis using PCT.

Regarding the use of PCT as an antibiotic guide: bacterial resistance to antibiotics has emerged as an important factor affecting the outcomes of infected patients. This has led to the need for major efforts to reduce the overuse of antimicrobials. Multiple clinical trials and meta-analyses have sought to use PCT levels as a guide to when to start, decrease, or discontinue the use of antibiotics in patients with suspected or diagnosed sepsis [47,48,49]. The results vary and are difficult to compare due to the heterogeneity of the patients (patients in CCU, emergency, respiratory infections, UTI, postoperative, etc.); however, the data seem to suggest that it is possible to reduce the use of antibiotics guided by PCT without increasing the morbidity and mortality of septic patients. In Table 3, we show the classic and still valid algorithm proposed by Schuetz et al., who recommended discontinuing the use of antibiotics in critically ill patients once PCT has normalized or, at least, when it has decreased by 80–90% of its peak value [50].

Significant PCT elevations can occur in high-stress situations; this is why PCT is more useful in medical than surgical patients to discriminate infection from sterile inflammation, as PCT tends to rise with surgery. This elevation depends on the site of intervention and the complexity of the technique performed. In addition to surgery, many other causes of PCT elevation due to nonbacterial systemic inflammation have been described [51]. In the case of fungal infections, slightly elevated or normal values have been observed in neutropenic patients with candidemia, so PCT has poor value for the diagnosis of fungal sepsis.

In the 2021 Surviving Sepsis Campaign guidelines [52], the two recommendations in relation to PCT are as follows:

- “For adults with suspected sepsis or septic shock, we suggest against using procalcitonin plus clinical evaluation to decide when to start antimicrobials, compared with clinical evaluation alone.” Quality of evidence: very low, weak recommendation.
- “For adults with an initial diagnosis of sepsis or septic shock and adequate focus control where the optimal duration of therapy is unclear, we suggest using procalcitonin and clinical evaluation to decide when to discontinue antimicrobials rather than just clinical evaluation.” Quality of evidence: low, weak recommendation.

Although there is no clear evidence for its use in diagnosis, PCT has represented a fundamental tool for early identification of patients who develop infection and for determining its clinical severity. Rapid identification of bacterial infections and early initiation of antibiotic therapy are recognized as independent factors associated with better outcomes; therefore, the immediate recognition of a bacterial infection through the use of a biomarker such as PCT can be very useful in situations of shock of doubtful infectious etiology that may be sepsis.

Recent studies have demonstrated the potential role of PCT in discriminating between serious infections caused by Gram-negative bacilli (GNB), Gram-positive bacilli (GPB), and fungi [53], especially in the context of bacteremia, where the level of PCT could be an important tool for quickly choosing the most appropriate antibiotic. In clinical practice, rapid identification of pathogens is often delayed due to available microbiological testing. The identification of etiologies, using biomarkers such as procalcitonin, can be very useful to avoid delays in treatment and inappropriate therapies.

GNB infections are associated with much higher levels of PCT than GPBs and fungi, which raise it less. Above all, Enterobacteriaceae are microorganisms that most elevate procalcitonin, in relation to endotoxin levels. Bassetti et al. proposed an algorithm of action based on procalcitonin levels to decide empirical antibiotic therapy according to procalcitonin levels; if they are greater than 2 ng/mL, they recommend thinking about GNB etiology, and if higher, they recommend thinking about Enterobacteriaceae [54]. This is an example of how knowing how a biomarker works in different contexts and patients, its kinetics, and everything that may affect it makes its use more effective and profitable.

### 2.6. MR-ProAdrenomedullin (MR-ProADM)/BioAdrenomedullin (BioADM): Biomarkers of Endothelial Dysfunction, Organ Failure, and Systemic Congestion

Adrenomedullin (ADM) is a peptide hormone isolated in 1993 by Kitamura from extracts of a pheochromocytoma [47]. Since this peptide is abundant in normal adrenal medulla, as well as pheochromocytoma tissue, it was called ADM. ADM possesses 52 amino acids, has an intramolecular disulfide bond, and shows slight similarity to the calcitonin gene-related peptide. The mRNA encodes the information for the synthesis of a preprohormone known as pre-pro-adrenomedullin, of 185 amino acids, subsequently degraded into another of 164 amino acids called ProADM. ProADM has three vasoactive peptides: ADM, proadrenomedullin amino-terminal peptide (PAMP), and adrenotensin [55]. There is also a middle region without activity, known as MR-proADM, which until recently was the most affordable for measurement. ADM occurs primarily in vascular endothelial cells. Among its fundamental biological actions are vasodilator, inotropic effects, diuretic, natriuretic, bronchodilator, insulin secretion inhibitor, aldosterone inhibitor, and adrenocorticotropic hormone inhibitor [56]. ADM, therefore, seems to function as a system that controls circulation and volume, and may be involved in cardiovascular pathophysiological changes. Its potent hypotensive and vasodilator activity depends on at least two mechanisms: a direct effect on vascular smooth muscle cells, increasing intracellular cAMP, and stimulation of calcium-dependent nitric oxide synthesis in endothelial cells. Plasma levels of ADM are elevated in cardiovascular diseases such as heart failure, hypertension, and septic shock, where ADM may play protective roles through its biological activities. Elevated levels are also found in other diseases such as heart failure, respiratory problems, kidney disease, liver cirrhosis, and cancer. High levels have been described in patients with sepsis, acting directly on the relaxation of vascular tone, triggering hypotension [57].

Krintus et al. established reliable ranges of MR-ProADM plasma values in healthy individuals, with the values for the 2.5th and 97.5th percentiles of MR-ProADM being 0.21 (0.19–0.23) and 0.57 (0.55–0.59) nmol/L, respectively [58].

Its pathophysiological action, added to the confirmation of its increase in the context of cardiovascular diseases, makes it a biomarker of cardiovascular status. Plasma levels of MR-ProADM increase in proportion to the severity of heart failure, which may reflect the fact that endothelial dysfunction is deeply involved in the pathophysiology of heart failure [59].

The Biomarkers In Acute Heart Failure trial [60] evaluated the clinical utility of MR-ProADM in heart failure. Patients who died had a higher median MR-ProADM than survivors (1.57 nmol/L vs. 0.84 nmol/L). Elevated MR-proADM levels predicted 90-day mortality in all patients with dyspnea and did so independently of natriuretic peptide concentrations. MR-ProADM, despite being a marker of organ failure (more cardiovascular than infection), has been widely studied in infection, in both diagnosis and prognosis. It has been studied in sepsis, with different cutoff values always higher than 1 nmol/L, to detect patients with poor prognosis. The determination of a cutoff point of MR-ProADM to predict organ failure and mortality was evaluated in patients with sepsis by Bernal-Morell et al. [61]. The cutoff point of MR-ProADM to detect organ failure in this context was 1.8 nmol/L, with a negative predictive value of 54% and a positive predictive value of 90%. Andaluz et al., in a study of ProADM in septic patients, concluded that an MR-ProADM value of 1.79 nmol/L predicted higher mortality [62]. The MR-proADM value less than 0.88 nmol/L in this study ruled out mortality in the 28 days after admission to CCU, since no patient with lower values died. There are few publications referring to the role of MR-ProADM as a risk marker in the perioperative context. The article by Schoe et al. investigated whether a set of biomarkers (PCT, MR-ProADM, CT-pro-endothelin-1, CT-pro-arginine-vasopressin, and NT-ProBNP), alone or as a panel, could be useful in assessing the postoperative risk of in-hospital mortality compared to the APACHE IV score [63]. It was found that patients with plasma levels of MR-ProADM of >3.2 nmol/L in the first 6 h postoperative had higher hospital mortality, showing greater predictive capacity than the APACHE IV scale (AUC 0.94 vs. 0.84). Csordas et al. investigated the predictive value of MR-ProADM mortality in a population of 153 patients scheduled for transcatheter aortic valve replacement [64]. MR-ProADM levels of >1.3 nmol/L were shown to be an independent predictor of mortality (31% vs. 4%, RR 9.9; CI 95% 3.1–31.3). In the perioperative context, we must mention the studies related to the determination of MR-ProADM for the diagnosis of acute appendicitis. Our group conducted a prospective observational pilot study at La Princesa University Hospital, which included a total of 59 adult patients scheduled for major abdominal surgery [65]. We studied whether preoperative levels of MR-ProADM could be predictors of the need for Postoperative Organ Support (POS) in these patients. For the association between MR-ProADM levels and POS incidence, an AUC-ROC of 0.85 was obtained (95% CI: 0.74–0.96; *p* = 0.002). In the multivariate analysis performed, preoperative serum MR-ProADM levels were an independent risk factor for the need for POS.

Finally, Bermejo et al. analyzed the ability of MR-ProADM and other markers to detect the different possible organ failures in patients with infection. In his research, the AUC-ROC of MR-ProADM for the diagnosis of organ failure was 0.79 (0.72–0.86), considered a very good predictive capacity, above that obtained with other biomarkers studied in the same work such as PCT: 0.62 (0.54–0.70), lactate: 0.69 (0.61–0.78), or CRP: 0.54 (0.45–0.63) [66]. Biomarkers could add valuable information to clinical judgment to detect the presence of organ failure early on during infection. MR-ProADM was the biomarker independently associated with the highest number of organ failures. Thus, MR-Pro-ADM could summarize the information provided by the six elements of the Sequential Organ Failure Assessment (SOFA) score. This probably explains why MR-ProADM was also the best biomarker predicting mortality.

Our group has conducted a multicenter study (preparing for publication and the subject of a doctoral thesis), whose results confirmed the association between preoperative serum levels of MR-ProADM and the need for POS in the first seven days after scheduled abdominal oncological surgery of intermediate and high risk. A total of 370 patients in four university hospitals were studied. The mean preoperative value of MR-ProADM was 0.81 ± 0.65 nmol/L, with a median of 0.66 nmol/L. The ROC curve was analyzed for association between preoperative values of MR-ProADM and the need for POS, obtaining an AUC-ROC of 0.67 (95% CI 0.59–0.75). The preoperative value of MR-ProADM, with a better sensitivity and specificity compromise to predict the need for POS, was 0.7 nmol/L. This is the first prospective, multicenter study to establish the prognostic value of MR-ProADM to predict the need for POS. It is interesting that a negative predictive value higher than 90% was obtained, allowing us to confirm, with high probability, that patients scheduled for abdominal oncological surgery with preoperative serum levels of MR-ProADM < 0.70 nmol/L will not require POS [67].

**BioADM** detects adrenomedullin directly, and until recently could not be measured by its kinetics. It is called BioADM because it detects biologically active ADM [54]. The normal value range of BioADM is 8–39 pg/mL. BioADM is an active molecule and is not influenced by inflammation compared to MR-ProADM, which has no known physiological function and is elevated in inflammatory states [68]. BioADM increases if endothelial function is severely impaired, and patients develop septic shock. It is a very sensitive marker of endothelial dysfunction: if the situation improves, the concentration of BioADM in the blood is rapidly reduced and the success of therapy can be monitored [69]. BioADM is a dynamic and specific marker to predict and monitor the evolution of septic shock. In specific studies of sepsis, the cutoff points of BioADM related to poor evolution range between 70 and110 pg/mL [70]. BioADM has been shown to be useful to guide the treatment of sepsis in multicenter prospective studies, with a threshold of >70 pg/mL being associated with worse results [71]. ADM is also a biologic target for the development of drugs to treat septic shock such as adrecizumab [72]. BioADM has begun to be studied in the perioperative setting in critically ill patients with sepsis after major surgery. Thus, Simon et al. found elevations of the plasma level of ADM in relation to the severity of patients with sepsis after major surgery: 16.2 pg/mL in the control group; 25.8 pg/mL in the sepsis group; 84.2 pg/mL in the severe sepsis group; and119.7 pg/mL in the septic shock group. A higher level of BioADM at admission was associated with a greater need for vasopressors and mortality [73]. Therefore, BioADM may be a useful additional parameter in surgical patients with sepsis. BioADM has been studied as a marker in the evaluation of venous congestion associated with heart failure and correlates very well with measures of systemic congestion and mortality in decompensated heart failure. ADM is released by endothelial and vascular smooth muscle cells in response to intravascular volume overload and plays a key role in maintaining endothelial barrier function, thereby regulating tissue volume and edema [74]. In fact, the role of biomarkers to evaluate pulmonary and systemic congestion and distinguish phenotypes has recently been highlighted [75]. Natriuretic peptides in pulmonary congestion and MR-ProADM and BioADM in both vascular and tissue congestion are very useful. Their elevated values are associated with the presence of edema, orthopnea, hepatomegaly, and high central venous pressure, being able to guide the decongestant and diuretic treatment. The usefulness of these biomarkers in congestion is an important field of research and development.

### 2.7. Neutrophil–Lymphocyte Ratio and Lymphopenia: The Markers in a Typical Analysis

The complete blood count has a long history in the diagnosis of septic shock. Despite its limitations, it is a pragmatic tool because patients will usually have a measured blood count upon admission to a hospital. Therefore, it is sensible to extract as much information from these values as possible [76]. Emerging evidence suggests that the emphasis should be on neutrophil–lymphocyte ratio.

The **neutrophil/lymphocyte index (NLR)**, defined as the ratio of the absolute neutrophil and lymphocyte count, is a marker of inflammation that is significantly associated with elevated levels of proinflammatory cytokines. Consequently, it may be a predictor of the development of sepsis, cardiovascular disease, or postoperative adverse outcomes. Unlike other biomarkers, NLR is a value that can be obtained economically and easily in routine blood tests. Physiological stress, cortisol, and catecholamines increase the number of neutrophils and decrease the number of lymphocytes, so the NLR will increase. Sepsis also stimulates lymphocyte apoptosis, so septic shock can cause a particularly dramatic elevation of the marker. NLR increases rapidly after acute physiological stress, often within 6 h and with a good outcome, and may consequently be useful in classifying patients with severe systemic diseases versus patients with milder diseases [77]. NLR usually begins to decline within a few days, and failure to improve over time correlates with a poor prognosis [78]. A normal NLR is about 1–3, and values increase in proportion to the degree of physiological stress, especially in septic shock. NLR has also been studied in the perioperative setting [79]. The neuroendocrine system is activated during anesthesia and surgery, resulting in the release of neuroendocrine hormones and cytokines and producing systemic leukocyte alterations including leukocytosis, neutrophilia and lymphopenia, lymphocyte apoptosis, or inhibition of neutrophil apoptosis. It has been proposed that the cutoff for the prediction of postoperative complications may be 5.5 and a preoperative NLR value ≥ 2.3 is associated with important postoperative complications in patients undergoing colorectal surgery [80].

**Lymphopenia:** An example of the usefulness of normal laboratory tests and related studies is the prognostic value of lymphopenia related to severe pneumonia. Bermejo et al. demonstrated (before the COVID-19 pandemic) that lymphopenia described an immunological phenotype associated with increased mortality risk in severe CAP. More than half of patients with severe pneumonia have fewer than 1000 lymphocytes per mm^3^, and those with lymphocyte counts below 724 per mm^3^ have a significantly increased risk of mortality at 30 days [81]. Lymphopenia gained relevance during the COVID-19 pandemic by identifying the patients with the most severe pneumonia. The presence of lymphopenia on admission and the absence of recovery to normal levels during treatment were related to mortality in SARS-CoV-2 pneumonia [82].

### 2.8. The Search for Markers of Renal Failure

Acute renal failure in the perioperative context and critical illness is relevant not only in terms of morbidity and mortality but also in terms of the need for resources and prolonged stays. Patients who suffer kidney failure associated with their surgical processes or critical illness have increased risk of mortality. They may also require renal replacement therapies, with the increase in cost that this generates, regardless of the severity of their condition [83]. Creatinine remains the biomarker of renal failure, being well related to morbidity and mortality, which allows for classifications and decision making. Creatinine theoretically estimates the glomerular filtration rate, although it is not filtered exclusively by the kidney and rises relatively late, after the onset of acute kidney injury (AKI) [84]. Therefore, more accurate and faster response biomarkers for AKI are required. Gold standard methods for determining glomerular filtration, such as inulin clearance or iohexole, are not feasible in acute clinical settings.

Thus, a relevant section of active research is the development of markers of renal function that allow for earlier decision making [85].

The characteristics of an ideal AKI biomarker are as follows [86]:

- Noninvasive and easily detected in accessible samples such as blood and urine;
- Their determination must be prompt and employ precise methods that are readily available;
- Very sensitive and specific to AKI;
- Useful in establishing the cause and mechanisms that lead to the development of renal aggression, as well as giving indications of the duration of the episode of AKI;
- Be early and detect minimal changes in glomerular filtration rate (even before depleting renal reserve and increasing serum creatinine value);
- Should not only indicate injury, but also alterations in kidney function;
- Be able to predict which patients will progress in the episode of AKI and who will be likely to recover;
- Have value for determining events such as the development of complications, need for dialysis, length of hospital stay, and mortality;
- Useful for targeting interventions that improve kidney function, in addition to monitoring course and response to established treatment;
- Their levels should not be affected by biological variability and systemic response;
- They should not be expensive, which would allow for universal application.

**Proenkephalin**: blood tests

Proenkephalin A 119–159 (PENK) has been intensively studied as a novel biomarker of renal function [87]. PENK belongs to the family of enkephalin peptides and is freely filtered into the glomerulus. Plasma PENK concentration appears to be strongly correlated with glomerular filtration rate, and increased plasma PENK concentrations are associated with long-term renal problems and mortality. Its elevation significantly anticipates the alteration of creatinine. It has been successfully studied in the context of renal failure of the critically ill patient [88]. Plasma PENK concentrations are measured using the penKid immunoassay at the NeXUS IB10 point of care. Values of more than 100 pmol/mL are related in sepsis to a glomerular filtration rate less than 30 mL/kg/1.73 m^2^ [89]. Renal replacement therapy (RRT) remains the key salvage therapy for critically ill patients with AKI. PENK has been studied in this context, resulting in levels of ≤89 pmol/L at the beginning of renal replacement therapy, but with a shorter therapy duration than in patients with higher values [90,91].

**Nefrocheck:** urine analysis

The NephroCheck^®^ assay is an in vitro diagnostic device that quantitatively measures the proteins TIMP-2 (tissue inhibitor metalloproteinase 2) and IGFBP-7 (insulin-like growth factor binding protein 7), related to renal function in human urine by a fluorescence immunoassay by the ASTUTE 140^®^ meter. TIMP-2 and IGFBP-7 molecules are produced in stressed kidney cells as an early warning signal, before the onset of acute renal failure, and are specific to renal stress, as they are not affected by any of the usual comorbidities such as sepsis, trauma, chronic kidney disease, or cancer [92]. The result is given in a single numerical value indicative of risk (“AKI Risk”), warning of the possibility of kidney damage with high sensitivity and a high negative predictive value. An AKI Risk value of ≤0.3 is indicative of a low risk of developing moderate or severe AKI within 12 h of assessment. A higher value indicates moderate or severe risk of kidney injury within 12 h; values greater than 2 double the possibility of adverse renal events and the need for RRT.

NephroCheck has been employed in intensive care and in the perioperative context for example in cardiac surgery [93]. Attempts have been made to associate its use with packages of renal protection measures when the value is high, an interesting strategy for the use of a biomarker associated with personalized management [94]. These strategies were useful for reducing renal failure, but only in those who had high values, demonstrating the need to determine in advance which patients could potentially benefit.

**Neutrophil gelatinase-associated lipocalin**: Can be detected in blood and urine, more commonly in urine

Neutrophil gelatinase-associated lipocalin (NGAL) is a small protein belonging to the lipocalin superfamily. NGAL is expressed at very low levels in different tissues, such as the kidney, trachea, lungs, stomach, and colon, and its expression increases markedly in inflammation. Therefore, it constitutes a biomarker of systemic leukocyte activation, being considered an acute phase reactant. Its specific function is not fully clarified, having described a renal protective role [95]. NGAL is freely filtered and reabsorbed at the proximal tubular level by endocytosis. Lesion of the proximal tubular epithelium alters its resorption. On the other hand, under conditions of renal damage, the expression of NGAL in the distal tubular epithelium increases, particularly in the ascending branch of the loop of Henle and in the collecting tubule.

The urinary concentration of NGAL increases under conditions of tubular damage, both by less reabsorption and by greater release into the tubular lumen, indicating both proximal and distal tubular damage. NGAL is an early marker of renal damage, since its serum concentration rises 2 h after damage and precedes by 24 h the increase in serum creatinine concentration. Their urinary and serum concentrations are also elevated in other conditions, such as UTI and chronic kidney disease. As it is a marker of kidney damage, it is known as renal troponin [96].

It has been studied in the perioperative context—for example, in cardiac surgery, relating very well its urinary and plasma concentration to the development of perioperative renal failure [97]. In more heterogeneous populations such as intensive care, there is also a relationship, but not as strong. NGAL levels are also elevated in sepsis and systemic inflammation, suggesting that its release into the urinary system is an important kidney response to systemic infection and local urogenital infection [98]. There are commercial kits to calculate uNGAL (urinary NGAL) or pNGAL (plasma NGAL). Under stable conditions, plasma and urine concentrations are around 20 ng/mL. The marker rises within 2–4 h of kidney damage. It has been suggested that uNGAL values <50 ng/mL indicate low risk of kidney damage. Between 50 and 149 ng/mL is a gray area that would involve repeated measurements and surveillance. Between 150 and 300 ng/mL, there is a risk of moderate kidney damage with high sensitivity, and over 300 ng/mL, there would be a high risk of damage with high specificity [95].

### 2.9. The Most Promising Current Biomarkers Available

The number of biomarkers does not stop increasing, as they are understood to be the best possibility of personalization and phenotyping. We have seen in detail some of the most accessible or the most studied. In this section, we will discuss some of the most interesting, with the possibility of using them today. We start from the premise that any choice on this topic is debatable, but being a narrative review, we choose those that we think can give the reader an overview of what will improve results. For the full picture, see below [99].

#### 2.9.1. Interleukin 6

Interleukins are cytokines released by multiple immune system cells such as monocytes, T cells, fibroblasts, and endothelial cells. They have numerous functions in different systems and organs and serve as intercellular communication carrying signals to neighboring cells to modulate and originate an immune response, producing inflammation against infection. Interleukin 6 (IL-6) is one of the most important biomarkers in sepsis. It has pleiotropic action—that is, pro-inflammatory and anti-inflammatory activity. Its actions on immunity are multiple and they affect the stress response, coagulation, and inflammatory response. The literature is very broad in linking IL-6 directly with the pathophysiology of several autoimmune diseases. There are antibodies used in treatment to inhibit the action of IL-6, as in the treatment of rheumatoid arthritis [100]. In COVID-19 patients, IL-6 levels are significantly elevated, so serum IL-6 was used as a biomarker of COVID-19 severity and to decide on the administration of immunosuppressive treatment for the famous cytokine storm, based on experience in rheumatological and autoimmune diseases. Subsequently, this approach has been qualified [101]. IL-6 levels rise after surgery, trauma, or critical illness. The magnitude of IL-6 elevation correlates with the extent of tissue trauma or severity of injury. In addition, there is an association between IL-6 elevation and adverse outcome. IL-6 levels can also be used to stratify patients for therapeutic intervention [102]. Thresholds vary between systemic inflammatory response syndrome, sepsis, and septic shock, with values of 40, 100, and 500 pg/mL, respectively [103].

#### 2.9.2. Urokinase-Type Soluble Plasminogen Activator Receptor (suPAR)

The urokinase-like soluble plasminogen activator receptor (suPAR) was first identified by Danish researchers in 1990 as a biomarker associated with cancer and its progression. Subsequently, it was associated with the prognosis of patients with bacterial and other infections, thus paving the way for its study as a biomarker in sepsis [104]. The suPAR biomarker is the soluble form of the cell membrane-bound uPAR protein, which is expressed primarily in immune cells, endothelial cells, and smooth muscle cells. suPAR is released in inflammation or immune activation and, therefore, the level of suPAR reflects the degree of immune activation in the subject [105]. Savva et al. published the first study determining the prognostic function of suPAR in patients with VAP in CCU, finding that values of >12.9 ng/mL corresponded to higher mortality at 28 days [106]. Since then, other studies have obtained similar results in sepsis and serious infectious pathology with thresholds of >12.9 ng/mL, proving it as an excellent prognostic biomarker in critical patients, but it does not discriminate those with sepsis from another type of pathology and therefore could be considered nonspecific at the time of its interpretation since suPAR is elevated in patients with cardiovascular, hepatic, renal, and pulmonary diseases as well as several infectious diseases [107]. Normal values for subjects aged 50–70 years are 3.0 ng/mL and rise with age. Patients admitted to CCU haven median values greater than 5.6 ng/mL. A suPAR level above 12 ng/mL in patients admitted to CCU is associated with increased mortality with a sensitivity of >80% [108]. In the surgical setting, a high level of preoperative suPAR is associated with a greater number of postoperative complications and an increased risk of mortality [109]. suPAR > 6 ng/mL is the cutoff to identify risk and possible serious disease [110].

#### 2.9.3. Presepsin

Presepsin is the soluble subtype of the CD14 or sCD14-ST glycoprotein expressed on the surface of monocytes and macrophages. CD14 is the receptor of protein-bound lipopolysaccharide complexes, which translates the signal of endotoxins released by GNB and other stimuli, through the Toll-Like receptor 4 (TLR4), leading to a cascade activation that gradually activates the transcription of nuclear factor kappa B, which leads to the release of cytokines [111].

Its elevation implies the activation of monocytes and macrophages by an inflammatory or infectious stimulus. It rises in the early stages of sepsis, 2 h after the inflammatory response starts, reaching its peak at 24 h. However, there may also be elevated levels in other inflammatory processes. Presepsin is available in the PATHFAST analyzer using a chemiluminescence test. Its preliminary values for sepsis are <200 pg/mL and the diagnosis of sepsis corresponds to >300 pg/mL [112]. In the perioperative setting, elevated presepsin is associated with major cardiovascular and perioperative cerebrovascular complications in high-risk patients undergoing noncardiac surgery [113]. The presepsin cutoff of 184 pg/mL could qualify to complement NT-proBNP-based risk prediction in perioperative high-risk patients, thereby increasing the proportion of correctly identified high-risk patients. It has also been proposed as a biomarker for predicting mortality in cardiac surgery [114].

#### 2.9.4. Pancreatic Stone Protein (PSP)

The first function described for pancreatic stone protein (PSP) was the inhibition of the growth of calcium carbonate crystals in pancreatic juice. PSP has also been associated with pathological changes that occur in the pancreas during pancreatic inflammation [115]. A fundamental observation was made by chance in experiments with rats by Grafs group, in which PSP turned out to be an indicator of systemic stress, as subsequently confirmed by numerous studies. It appears that the pancreas senses remote organ damage and systemic stress and responds by secreting PSP, particularly when associated with serious infectious complications and sepsis, as PSP could activate neutrophils and promote bacterial aggregation [116]. Normal PSP values in healthy volunteers are 10.4 ng/mL (7.5–12.3). PSP is a promising biomarker for early diagnosis of infections in hospitalized patients using a cutoff value of 44.18 ng/L [117]. Its value can be obtained in a point of care Platform Abioscope^®^. In several scenarios, such as trauma and cardiac surgery, its elevation predicted the onset of sepsis before it occurred clinically [118]. PSP can also help stratify patients according to their severity [119].

#### 2.9.5. Dipeptidyl Peptidase 3 (DPP3)

Dipeptidyl peptidases are a class of proteolytic enzymes involved in almost every aspect of cellular activities and physiological functions. Dipeptidyl peptidase 3 (DPP3) is an active enzyme that, when released into the blood, inactivates angiotensin II, a hormone that is key to hemodynamic balance and heart function. This inactivation leads to hemodynamic instability and, consequently, cardiac dysfunction. DPP3 release is a newly identified disease mechanism that explains short-term organ failure in critically ill patients. Early identification of DPP3 release may allow for better patient stratification and earlier escalation of therapy to improve outcomes. Circulating DPP3 is a myocardial depressant factor. High DPP3 levels have been associated with reduced cardiac output, multi-organ failure, and circulatory shock [120]. It has been observed that blood levels of DPP3 in septic shock are elevated and that low or decreasing levels of DPP3 in the first 24 h of admission to ICU predict improved organ function and better outcomes. Conversely, elevated blood levels precede organ failure and predict the need for vasopressor or inotropic drug use, mechanical ventilation, renal failure, and short-term mortality [121]. The cutoff values for DPP3 are 33–40 ng/mL, and it is available at a SphingoTec point of care. Nexus IB10. In patients with PDP3 > 40.4 ng/mL on admission, in whom a decrease in its value was observed in the first 24 h, it is associated with an improvement in organ function. Higher DPP3 concentrations were associated with more pronounced cardiovascular dysfunction, such as the need for vasopressor therapy.

Given its hemodynamic effects, DPP3 has been considered as a biomarker of shock and, hopefully, much more so since its reduction by treatment has been shown to substantially improve hemodynamics and outcomes [122]. Anti-DPP3 therapy already exists that restores heart function and hemodynamic stability and improves survival. The anti-DPP3 drug candidate antibody, Procizumab, has already demonstrated efficacy in preclinical models and will enter the first human clinical trial in late 2022 [123]. It is a very promising biomarker not only for diagnosis and stratification, but also for guiding hemodynamic and shock therapy [124].

### 2.10. The Future Outlook

There are numerous biomarkers, and their positioning will need to be validated [125,126]. However, precision medicine seeks the precision of treatments and their personalization and, as has already happened in oncology, in critical and perioperative medicine this grail is being sought to improve results [99]. In this sense, the panels of biomarkers, the association of biomarkers with scales, the use of the point of care of biomarkers, the development of therapies specifically designed to control biomarkers with biological effects that condition the results, and the development of systems biology and genomics will improve the accuracy, speed, and efficiency of patient care.

**Point of Care (PoC)** is becoming more frequent in the perioperative context and ICU. The most common equipment is that for blood gas, hematimetry, and basic biochemistry, as well as for coagulation tests. There is interest in providing cost-effective biomarkers in PoC because of the speed of obtaining results at the discretion of the clinician at the time needed. A PoC biomarker must be affordable, sensitive, specific, easy to use, fast, robust, and effective [127].

Being able to have a PoC in the surgical area and ICU that provides reliable values of biomarkers would result in relevant information on high-risk patients, and could improve or complement the information provided by the usual and exceptional analytical, clinical, and monitoring variables [126]. The precision medicine towards which medical practice is heading, also in the perioperative and critical medicine context, makes point of care increasingly common as technology improves and research evolves. Many of the biomarkers we have seen in this review have an associated point of care. In some cases, such as Nefrocheck or PSP, they have the possibility of their own biomarker; or others, such as NEXUS IB10, offer the possibility of having numerous biomarkers such as NT-ProBNP, Troponin, DPP3, BioADM, etc., using different discs for each. The possibility of having biomarkers at the bedside, combined with the best knowledge and use of the means usually available, will undoubtedly improve the speed and accuracy of care [128].

The development of therapies specifically designed to control biomarkers with biological effects has already arrived. The non-neutralizing anti-Adrenomedullin antibody Adrecizumab has shown promising results in animal models of systemic inflammation and sepsis, and in a phase II human trial. It stabilized the endothelium, reduced inflammation, attenuated vascular leakage, and improved hemodynamics, kidney function, and survival, with an excellent safety profile derived from phase I animal and human studies. Adrecizumab represents a promising drug candidate for the adjuvant treatment of sepsis [72]. Procizumab is a humanized monoclonal antibody in preclinical development that specifically binds to circulating DPP3. It aims to modulate DPP3 as an essential regulator of cardiovascular function. Procizumab inhibits DPP3 activity, which reduces bioactive peptide degradation, stabilizes hemodynamics, cardiovascular function, and potentially increases the chances of survival, for example, in shock.

Preclinical studies of Procizumab have demonstrated efficacy, leading to validation in clinical trials [123].

### 2.11. A Systems Biology Approach

The combination of technological advances and information generated through the Human Genome Project positions systems biology at the forefront of biomarker discovery. While previously available, advances in DNA-centric technologies, gene expression, gene regulatory mechanisms, and protein and metabolite discovery have made these tools more feasible to implement in the near future. Genomics is the study of the entire complement of genetic material of an individual. Epigenetics is the regulation of gene activity by reversible modifications of DNA. Transcriptomics is the quantification of the relative levels of messenger RNA for a large number of genes in specific cells or tissues to measure differences in the expression levels of different genes and the use of differential gene expression patterns to characterize different biological levels of a tissue. Proteomics is the large-scale study of proteins. Metabolomics is the study of the profiles of small molecules that are the end products of the genome and consists of the total complement of all the molecules of low molecular weight that leave the cellular processes. Together, these individual fields of study can be linked in a systems biology approach. Omics technologies may improve precision medicine in the future [129]. In the case of human genetics, the main focus in relation to disease is to analyze genetic variations called single-nucleotide polymorphisms (SNPs). The Genome-Wide Association Study simultaneously probes all segments of the genome for evidence of association between a known SNP and disease, comparing sick and well populations to identify the SNPs that are most prevalent in disease status [130].

MicroRNAs (mRNAs) are small single-stranded RNAs that do not code for proteins. They function as post-transcriptional regulators of gene expression by interacting with target mRNAs. It is considered that mRNA is functionally involved in virtually all physiological processes, including differentiation and proliferation, metabolism, hemostasis, apoptosis, and inflammation. Many of these functions have important implications for anesthesiology and critical care medicine. The expression levels of mRNA could be used to predict the risk of sepsis or organ injury [131].

## 3. Biomarkers in High-Risk Perioperative Patients

Most perioperative risk guidelines include biomarkers for the assessment of high-risk patients (Table 4) [32,132,133].

The most recommended biomarkers are troponin and natriuretic peptides. Despite their recommendation in the guidelines, they are not widely used in most anesthesiology and perioperative CCU. In our field, we have used natriuretic requests successfully for some time, following the guidelines.

Our model is the Canadian guidelines of the Devereaux group, in which it is considered that patients at risk should be stratified and that natriuretic peptides do so effectively, allowing the use of resources such as monitoring, perioperative ultrasound, admission to CCU, and use of inotropics to be based on objective data [32]. Cutoff points are accepted and are BNP greater than 92 mg/L and NT-ProBNP greater than 300 mg/L.

With troponins, despite being a more accessible biomarker than natriuretic peptides, the use is not widespread despite ample evidence. Perhaps the reason is the different troponins and the fact that the effects are sometimes more long-term, so not seen in the immediate perioperative period. It seems that the spectrum of myocardial damage and elevation of troponins is related to several pathologies, and this lack of specificity can make its use less effective; however, a recommendation may be for its pre- and postoperative use because its modification can better identify those patients at high risk.

The latest guidelines, published in 2022 by the European Cardiology Association (ESC) with the support of the European Society Anesthesiology and Intensive Care (ESAIC) [133], can help us with these difficult situations. They propose that underlying heart disease is important for the results and biomarkers can detect it as hsTnT or hsTnI, which quantifies myocardial injury, and BNP and NT-proBNP, which quantify the hemodynamic stress of the heart wall. Both hsTnT/I and BNP/NT-ProBNP complement clinical and ECG assessment in risk prediction, and troponins have a very high negative predictive value to rule out myocardial damage.

ESC guidelines recognize that there is important evidence from large prospective studies that have shown that both hsTnT/I and BNP/NT-ProBNP have a high and increasing prognostic value for perioperative cardiac complications—in the case of ProBNP, even surpassing both specific risk scales and echocardiographic evaluation.

Although the ESC guidelines recognize the similar prognostic yield of both biomarkers, they lean more towards troponins due to four advantages that they have over BNP/NT-ProBNP:

1. Troponin is more available.
2. Troponin is less expensive.
3. If normal, troponin allows one to rule out acute myocardial infarction.
4. If the preoperative concentration of hs-cTn T/I is available, it allows for an accurate diagnosis of perioperative myocardial infarction on day 1 after surgery thanks to the comparison with the postoperative values.

The ESC guidelines also recognize the merits of natriuretic peptides, as they allow for the detection of patients with occult heart failure, especially the elderly, which can improve their maintenance and perioperative treatment, and which allows one to guide the therapy of perioperative heart failure with better targeted treatment.

They do not mention other biomarkers, but there are some not included in the guidelines that may be very useful in the future, as we have seen in the perioperative context. We would highlight MR ProADM and Bio ADM since they have already been studied in the perioperative period [134], and their use as a marker of endothelial dysfunction [135] and congestion [136] makes them very attractive in this context.

Also, the markers of renal function, especially in cardiac surgery, have been studied and their interpretation is easy.

Finally, the highlight in innovation is DPP3, for its possible unique ability to identify patients at risk of organ failure [121], especially hemodynamic failure with the need for vasopressors [123].

## 4. Biomarkers in Sepsis and Intensive Care

In this review, we have discussed the use of numerous biomarkers that can be used in intensive care. Many of them are perhaps somewhat nonspecific and, although they evaluate severity well, may not be as useful to show the evolution and response to treatment. Perhaps better are those that, in addition, are more specific for use as a target in the sense that their reduction is a therapeutic objective associated with the improvement of results, as already happens in a clear way with procalcitonin, lactate, natriuretic peptides, and Bio ADM or MR-ProADM [137]. One more step would be to use them as treatment targets, as we have seen with BioADM and DPP3 [138].

There is a debate in intensive care about the use of biomarkers. A lot is required of them, that they be “magic bullets” to guide treatment, but everything requires knowledge [139].

In the case of biomarkers, in addition to the characteristics that we have already mentioned and that we will now develop in the context of intensive care, knowledge of their kinetics and characteristics is required. An example is the elevated levels of PCT that may suggest Enterobacteriaceae bacteremia [54].

One should ask the following questions prior to use in ICU [4]:

✓ What is the pre-test probability for the diagnosis? That is, is the test necessary or do I already have the diagnosis without needing it?
✓ Are there factors present that interfere with the interpretation of the result of the biomarker? As we have seen, age, comorbidity, kidney failure, etc. significantly affect the levels of most biomarkers and blur their specificity in some contexts.
✓ Will I change management depending on the result of the biomarker? In our opinion, this is a key question and defines the usefulness of a biomarker for use in a unit. This answer will influence the available evidence and one’s knowledge about it.
✓ Will the outcome of biomarker-guided decisions be beneficial? This is the holy grail question: if the answer were positive, doubts would not exist, but in the context of critical medicine, there are few things that can be answered categorically in the affirmative.

On the contrary, we have evidence that many biomarkers are already useful. It cannot be doubted that there have been enormous advances and that in pathologies such as severe infection, severe CAP, VAP, and sepsis, biomarkers have contributed a lot in management, as well as in acute heart failure and other critical pathologies. There are fields such as congestion, relevant in recent years, in which biomarkers can play a key role not only in their phenotypic diagnosis, but also in therapeutic guidance [75].

There are other occasions in intensive and perioperative care where biomarkers are useful, such as nutrition, and that we have not been able to address in this review due to its length [140]. Our group has also carried out research on the subject, validating the use of the CONUT tool based on the levels of albumin, cholesterol, and lymphocytes to evaluate the nutritional level [141]. A relevant aspect is sustainability, and it is our duty to confirm the feasibility, clinical impact, and economic benefit of the measurements made with any new biomarker that we want to incorporate into clinical practice.

In our view, this guiding aspect of treatment is key. One more step will be the biomarker as a treatment target. It is impressive how the times for the development of new biomarkers and targeted therapies are much shorter now than in the past. Many developments are waiting for us in this field; however, the excess supply of biomarkers can cause confusion [125]. Each unit, depending on its type of patients, experience, and resources, should choose the biomarkers that are best suited to its clinical practice, including as a point of care.

Biomarkers are the spearhead in ICU for precision medicine, and their role is still to be clearly defined in the coming years [142]. Its development parallel to clinical phenotyping, the development of systems biology, artificial intelligence, and big data, are the future challenges that precision medicine holds for us [143].

## 5. Conclusions

Biomarkers are an important piece of precision medicine because they allow establishing prognoses that stratify risk and a better use of resources in intensive and perioperative care. The ideal biomarker that is also reliable should help improve results. The AUC ROC is the appropriate statistical test to assess a biomarker. There are numerous biomarkers of possible interest; some are very integrated into our clinical practice and others have only been postulated with few validations or clinical utility. It is necessary to emphasize the present usefulness of the biomarkers in the field of infection and congestion in the critically ill patient, and in the stratification of perioperative risk, among others, with biomarkers of clinical utility that can improve the results.

In the near future, we will have therapies specifically associated with deficits of certain biomarkers, and the biomarkers will allow to describe phenotypes directly associated not only with prognosis but with the usefulness or not of certain therapies.

It is very important that clinicians know the advantages and limitations of biomarkers in intensive care, and their diagnostic characteristics for a rational and effective use of them. The new more specific biomarkers, the point of care of biomarkers, and the panels of biomarkers associated or not with clinical or genetic data, will set the course of prognosis in intensive and perioperative care in the coming years.

## Figures and Tables

**Table 1:** Biomarkers: main characteristics and cutoff points.

Biomarker	Characteristic	Cutoff	Considerations
**Lactate**	A perfusion marker	2 mmol/L	Beware of using clearance as a short-term therapy guide (hours)
**CRP**	A marker of inflammation	5 mg/dL	It is nonspecific for inflammation. A follow-up is helpful.
**Troponin**	A marker of myocardial damage	cTnT: 40 ng/L cTnI: 34 ng/L hsTnT: 14 ng/L	MINS is a spectrum of injury. The elevation of hsTnT between preoperative and postoperative greater than 14 ng/L is significant for the risk of cardiovascular complications and others such as sepsis.
**NT-ProBNP/BNP**	A marker of heart and lung congestion	NT-ProBNP: 300 pg/mL BNP: 30–50 pg/mL	Allows for monitoring of therapy for decongestion and treatment of cardiac dysfunction.
**Procalcitonin**	An infection marker	0.05–0.09 ng/mL	Useful to differentiate GNB from GPB and viruses, and to identify bacteremia (more than 2–4 ng/mL may be BGN). Useful in de-escalation of antibiotic therapy.
**MR-** **ProADM/BioADM**	A marker of organ failure and systemic congestion	MR-ProADM: 0.57 nmol/L. MR-ProADMin sepsis: NPV greater than 90% if it is less than 0.88 mmol/L. There is postoperative organic failure if it is greater than 0.70. BioADM:8–39 pg/mL. In sepsis, values higher than 70–110 pg/mL are related to organ failure and mortality.	Endothelial dysfunction marker. Allows for monitoring of organic failure and systemic decongestion. It is validated in sepsis and now also in the perioperative period. A drug has been developed against the elevation of BioADM: Adrecizumab
**NLR/linfopenia**	A usual analytical marker	NLR: 1–3 Lymphocytes: 4000–10,000/mm^3^	It implies severity of inflammation and is related to organ failure.
AKImarkers. **Proenkephalin**	Glomerular filtration marker in blood analysis	Values of more than 100 pmol/mL are related in sepsis with aglomerular filtration rate less than 30 mL/kg/1.73 m^2^	It is more specific, faster, and more useful than creatinine. Allows one to assess and predict the evolution of renal failure.
AKImarkers. **Nefrocheck**	Kidney damage marker in urinary analysis	The result is given in a single numerical value indicative of risk (“AKI Risk”); an AKI risk value ≤0.3 is indicative of low risk.	Measures the proteins TIMP-2 (tissue inhibitor metalloproteinase 2) and IGFBP-7 (insulin-like growth factor binding protein 7).
AKI markers. **Neutrophil gelatinase-associated lipocalin(NGAL)**	Tubular lesion marker Analysis possible in blood and urine	Under stable conditions, plasma andurine concentrations are around 20 ng/mL. >300 ng/mL, there would be a high risk of damage with high specificity	As it is a marker of kidney damage, it is known as “renal troponin”
**Interleukin 6**	Immunity, stress response, coagulation, and inflammatory response marker	Thresholds vary between systemic inflammatory response syndrome, sepsis, and septic shock with values of 40, 100, and 500 pg/mL	It has been used to direct therapy in the treatment of SARS-CoV-2. It has been used for phenotype differences in pathologies.
**Urokinase-type soluble plasminogen activator receptor (suPAR)**	Immunity and infection marker	Normal value for subjects aged 50–70 years is 3.0 ng/mL. suPAR > 6 ng/mL is the cutoff to identify risk and possible serious disease.	Its main use has been in the context of severe infection.
**Presepsin**	Its elevation implies the activation of monocytes and macrophages by an inflammatory or infectious stimulus.	Values for exclusion of sepsis correspond to values < 200 pg/mL and the diagnosis of sepsis is in a range >300 pg/mL.	It has been used in sepsis and in the perioperative period of major surgery to stratify and predict severity.
**Pancreatic Stone Protein (PSP)**	Stress and infection response marker	Normal PSP values are 10.4 (7.5–12.3) ng/mL. Early diagnosis of infections in hospitalized patients using a cutoff value of 44.18 ng/L.	It has been used to predict the occurrence of infections and sepsis in various ICU situations.
**Dipeptidyl peptidase 3 (DPP3)**	It is a marker of hemodynamic failure and cardiovascular dysfunction.	The proposed cutoff values for DPP3 are 33–40 ng/mL.	Higher DPP3 concentrations were associated with more pronounced cardiovascular dysfunction, such as the need for vasopressor therapy in septic shock and cardiogenic shock. There is an anti-DPP3 drug antibody, Procizumab

CRP: C-Reactive Protein; cTnT: Troponine T; cTnI: Troponine I; hsTnT: high-sensitivity troponine; MINS: Myocardial Injury after Noncardiac Surgery; GNB: Gram-Negative Bacteria; GPB: Gram-Positive Bacteria; NPV: Negative Predictive Value; NLR: Neutrophil Lymphocyte Ratio; AKI: Acute Kidney Injury; PSP: Pancreatic Stone Protein; ICU: Intensive Care Unit; DPP3: Dipeptidyl peptidase 3.

**Table 2:** PCT references values and their interpretation in sepsis.

PCT Concentration (ng/mL)	Interpretation	Recommendation
<0.05	Healthy individual (except neonates <48 h of life)	
<0.5	Possible but unlikely local infection	
0.5–2	Possible infection. Rule out other causes of PCT elevation (cardiogenic shock, trauma, surgery, etc.)	In case of proven infection, diagnosis of sepsis very likely. Monitor PCT every 6–24 h.
2–10	Sepsis very likely	High risk of organ dysfunction. Monitor PCT every 24 h.
≥10	Almost exclusively associated with sepsis	Very often associated with organ dysfunction. High risk of mortality. Monitor PCT every 24 h.

**Table 3:** Algorithm to guide antibiotic therapy in CCU in patients with suspected sepsis (reevaluation every 1–2 days).

PCT Result (ng/mL)	<0.25 or Drop by >90%	<0.5 or Drop by >80%	≥0.5	>1
Recommendation regarding use of Ab	Cessation of Ab strongly encouraged	Cessation of Ab encouraged	Cessation of Ab discouraged	Cessation of Ab strongly discouraged
Overruling the algorithm	Consider continuation of Ab if patients are clinically unstable	Consider continuation of Ab if patients are clinically Unstable	Consider continuation of Ab if patients are clinically unstable	Consider continuation of Ab if patients are clinically unstable
Follow up/other comments	Clinical reevaluation as appropriate	Clinical reevaluation as appropriate	Consider treatment to have failed if PCT level does not decrease adequately	Consider treatment to have failed if PCT level does not decrease adequately

PCT: Procalcitonin; Ab: Antibiotics.

**Table 4:** Summary of recommendations from the European Society of Anesthesiology and Intensive Care (ESAIC), the European Society of Cardiology (ESC), the Canadian Cardiovascular Society Guidelines (CCSG), and the American Heart Association (AHA).

ESAIC/ESC	CCSG	AHA
In patients who have known CVD, CV risk factors	If a patient’s age is ≥65, RCRI ≥ 1 or aged 45–64 with	High-risk individuals (i.e., >65 or >45 with
(including age ≥ 65 years), or symptoms suggestive of CVD, it is recommended to measure hs-cTn before intermediate and high risk NCS, and at 24 h and 48 h afterwards. In patients who have known CVD, CV risk factors (including age ≥ 65 years), or symptoms suggestive of CVD, it should be considered to measure BNP or NT-proBNP before intermediate and high risk NCS.	significant CVD, order BNP or NT-proBNP. AND if positive NT-proBNP ≥ 300 pg/mL or BNP ≥ 92 pg/mL OR BNP or NT-proBNP not available, THEN Measure troponin daily × 48–72 h Not routine hsTn monitoring if proBNP < 300 pg/mL	established CVD or peripheral atherosclerotic), having NCS, have serial hsTn measurements during the first 48–72 h postoperatively while hospitalized. MINS diagnostic criteria should be used to standardize assessment and reporting of ischemic events in clinical practice and future clinical trials

CVD: cardiovascular disease; NCS: noncardiac surgery; RCRI: revised cardiac risk index.
